# Childhood adversities, parental education and disability retirement among Finnish municipal employees

**DOI:** 10.1371/journal.pone.0219421

**Published:** 2019-07-19

**Authors:** Aino Salonsalmi, Olli Pietiläinen, Eero Lahelma, Ossi Rahkonen

**Affiliations:** Department of Public Health, University of Helsinki, Helsinki, Finland; Universidade Federal de Pelotas, BRAZIL

## Abstract

**Background:**

There is increasing evidence that childhood socioeconomic position and childhood adversities influence adult health. However, the potential contribution of these factors to disability retirement is poorly understood. This study aimed to examine the associations between childhood adversities, parental education and disability retirement.

**Methods:**

Data on parental education and childhood adversities were derived from the Helsinki Health Study baseline survey, conducted in 2000–02 among 40- to 60-year old employees of the City of Helsinki, Finland. Data on disability retirement and their diagnoses were obtained from the Finnish Centre of Pensions and followed until the end of 2016. The analyses included 5992 employees. The associations of parental education and childhood adversities with disability retirement due to any cause, musculoskeletal diseases and mental disorders were analysed using Cox regression analysis.

**Results:**

Low parental education was associated with an increased risk of disability retirement due to any cause (maternal education: HR 1.74, 95% CI 1.16–2.62; paternal education: 1.86, 1.38–2.51) and due to musculoskeletal diseases (maternal education: 4.44, 1.66–11.92; paternal education: 3.81, 2.02–7.17). However, adjustment for own education mainly abolished the associations. Economic difficulties in the childhood family, parental alcohol problems and having been bullied at school or by peers increased the risk of disability retirement due to all studied diagnostic groups, whereas parental death or divorce had no effect. Childhood illness (1.53, 1.20–1.95) and parental mental illness (1.68, 1.28–2.20) were associated with disability retirement due to any cause and due to mental disorders (1.65, 1.05–2.59; 3.60, 2.46–5.26). The associations between childhood adversities and disability retirement remained after adjustment for own education, whereas working conditions, and weight and health behaviours somewhat attenuated the associations.

**Conclusions:**

Parental education and childhood adversities contributed to disability retirement even in midlife. Policy actions investing in children’s well-being might promote work ability in midlife.

## Introduction

According to Finnish retirement schemes, disability retirement can be granted when an employee is unable to work as a result of reduced work ability because of ill health. Disability retirements have decreased during the past few years in Finland, but a quarter of all retirees, i.e. nearly 20 000 employees, still retire due to disability each year [1]. Work disability is also a major concern in other developed countries [2]. Thus, it is important to promote work ability and lengthen work careers. In order to do this a better understanding of the underlying mechanisms of disability retirement is required.

Evidence that childhood is associated with adulthood health [3–10] and mortality [11–12] is increasing. As a diagnosed disease is a prerequisite for receiving disability retirement childhood factors may also be behind disability retirement. Musculoskeletal diseases and mental disorders are the leading causes of disability retirement in Finland followed by neoplasms, neurological diseases and diseases of the circulatory system [1]. Childhood has been associated with diseases of all these disease groups in previous research [4–10]. The pathways between childhood and adulthood health are, however, complex. The critical period model suggests that biological and social conditions at vulnerable time periods may have long-term effects on health, whereas the accumulation of risk model suggests that ill health is a product of factors acting throughout the life course [13]. Previous studies have mainly examined childhood socioeconomic position, often defined by father’s occupational status. Childhood adversities such as parental divorce, parental alcohol consumption or mental illness have been studied less but have shown associations with adult health in previous research [14].

Research on childhood and disability retirement has been rare and to our knowledge no theoretical model exists. Firstly, the most direct pathway between childhood and disability retirement might be childhood illness or an accident affecting adult health and thus disability retirement. Secondly, childhood might contribute to adult health via adult education, adult marital status or adult health behaviours that are known determinants of health. For example, childhood has been associated with smoking, alcohol consumption, diet, obesity, and physical activity [15–16], all of which in turn have been associated with disability retirement [17–18]. A third potential pathway from childhood to disability retirement does not depend on health. In addition to health status, disability retirement is influenced by various other factors such as socioeconomic position, education and working conditions [15, 19–20], as well as behaviours and attitudes that might be influenced by childhood [21]. For example, socioeconomic inequalities in disability retirement are well known, and childhood factors have been also found to be associated with adulthood education [22] and income [23]. The pathways from childhood to disability retirement might be different across different diagnosis groups.

Previous studies on the association between childhood and disability retirement are scarce and have seldom used register-based data on disability retirement. In addition, these studies have focused on disability retirement at a relatively young age, namely under the age of 45. Of these studies, a Swedish study of men during mandatory military service reported that father’s occupational position, not being brought up by both parents, dealings with the police or child authorities, and remedial class were associated with all-cause disability retirement. It also found that father’s occupational position, remedial class and dealings with the police or child welfare authorities were associated with alcohol-related disability retirement [24]. Another study of the same data examined psychiatric diagnoses and found that many childhood adversities were associated with any psychiatric diagnosis, psychosis diagnosis and alcohol-related diagnosis [25]. A Norwegian study based on registers of childhood and disability retirement reported that the mother being unmarried when the child was born, parental disability and receipt of childhood disease benefit increased the risk of all-cause disability retirement, whereas parental education only had an effect on low-educated men [26]. A Finnish register-based study found that parental receipt of income support and care placement in adolescence increased the risk of disability retirement in early adulthood [27]. Another Finnish study reported that parental divorce, parental receipt of social assistance, mother’s and father’s psychiatric care and mother’s psychiatric disability retirement were associated with an increased risk of disability retirement due to mental disorders before the age of 25 [28].

However, disability retirement is more common among older employees and the determinants for disability may differ from those of the young, but studies of older employees are scarce. A Finnish study of self-reported data on disability retirement reported that an accumulation of childhood adversities increased the risk of self-reported disability retirement at midlife even after adjustment for adult socioeconomic position, adulthood somatic health and health behaviours [29]. Another Finnish study examined 19- to 68-year-old employees and found that the risk of work disability was higher among employees with childhood adversities only, with low adult socioeconomic position and with both exposures [15]. However, the outcome included both sickness absence and disability retirement and over 90% of work disability periods were due to sickness absence. Studies of a UK cohort with self-reported data on employment status at the age of 55 found that childhood adversities, psychological symptoms reported by a teacher, and neglect were associated with permanent sickness or work disability, whereas parental occupational class was not [30–32]. Studies of midlife employees have lacked register-based data on disability retirement and have not examined the diagnoses behind disability retirement.

The aim of this study was to examine whether parental socioeconomic position and childhood adversities are associated with disability retirement in midlife. Pathways from childhood to midlife disability retirement are examined by analysing whether own education, marital status, working conditions and adult weight and health behaviours contribute to the associations. All-cause disability retirement and the most common diagnostic groups behind disability retirement, namely musculoskeletal and mental diagnosis, are analysed separately.

## Data and methods

### Study population

The study is part of the Helsinki Health Study of employees of the City of Helsinki [33]. The City of Helsinki is the biggest employer in Finland, with about 37 000 employees, of whom 76% are women [34]. The City of Helsinki is responsible for diverse fields of activity such as social and health services, education, city planning, technical services, and art and culture. It has both white- and blue-collar occupations, for example, nurses, doctors, teachers, secretaries, garden workers, bus drivers, fire fighters, lawyers, and daycare workers.

The baseline survey was conducted in 2000, 2001 and 2002 by sending postal questionnaires to the City of Helsinki employees who turned 40, 45, 50, 55 or 60 during each year. A total of 8960 employees of the targeted 13 346 participated, yielding a response rate of 67%. The questionnaire included questions on socio-demographic factors, childhood, working conditions, health behaviours and health. Seventy-four per cent of the baseline participants consented to having their questionnaire data linked to data on disability retirement derived from the Finnish Centre for Pensions. Not all the participants answered all the questions, and after exclusions due to missing data on covariates (n = 489), the data consisted of 5992 employees. Of the participants, 1312 were men and 4680 (78%) were women, reflecting the gender distribution of the Finnish municipal sector. Disability retirement events were followed from the day of returning the baseline questionnaire until the end of 2016, the onset of retirement, the employee reaching the age of 63 or death; whichever came first. The mean follow-up time was 10.6 years.

Examination of the non-response of the Helsinki Health Study found that the response rate at baseline tended to be lower among younger employees, among those with lower occupational positions and among those with longer sickness absence during the study year [33]. Regarding consent to data linkage, men in lower occupational positions and to some extent women and men with medically certified sickness absences were overrepresented among the non-consenters. However, the non-response analyses suggested that non-response does not cause major bias [35].

The Ethics Committee of the Department of Public Health at the University of Helsinki and the Ethics Committee of the health authorities at the City of Helsinki approved the study.

### Parental education and childhood adversities

Data on parental education and childhood adversities were derived from the baseline questionnaire. The questions covered maternal and paternal education, offering six response alternatives. Parental education was divided into three classes: ‘Low’ (elementary school or part of it or intermediate school), ‘mid-level’ (vocational school or matriculation or college-level training) and ‘high’ (polytechnic or university degree). Another question inquired about adversities before the age of 16. Serious or long-term illness during childhood, parental divorce, parental death, father’s/mother’s mental health problems, father’s/mother’s alcohol consumption that caused problems at home, significant economic difficulties in the family, and being bullied at school or by peers were inquired. The childhood adversity variables had two response alternatives (yes/no) and those with no adversity served as reference categories.

### Disability retirement

Finnish 18–62-year-old municipal employees are entitled to disability retirement when they are unable to continue in their current job due to disease and their work ability cannot be restored by rehabilitation. To be eligible for full disability retirement, work ability must be reduced by at least 60% for a continuous period of at least one year. A medical statement including a medical diagnosis is required. [36]

The data on disability retirement included the main diagnoses for disability retirement according to the ICD-10 classification. There were 771 disability retirement events during the follow-up. In addition to all-cause disability retirement, the two most common disability diagnosis groups, namely disability retirement due to musculoskeletal diseases (ICD-10 codes M00-M99) and mental disorders (ICD-10 codes F00-F99) were analyzed. There were 324 disability retirement events due to musculoskeletal diseases and 212 due to mental disorders. Other diagnoses were rather heterogeneous and statistical power was insufficient to perform the analyses separately for these.

### Covariates

The data on covariates derived from the baseline survey. Sex was included as a covariate. Marital status was classified as single, married or cohabiting, or divorced or widowed. Educational attainment was divided into three classes: elementary school or intermediate school; vocational school, matriculation examination or college-level training; and polytechnic or university degree. Physical and mental working conditions were each measured using a single-item question with four response alternatives inquiring how physically and mentally strenuous the respondent considered their work. The response alternatives were divided into two classes: ‘Heavy’ and ‘light’. Weight was indicated by self-reported body mass index, which was divided into three groups: under 25, between 25 and 30, and above 30 kg/m^2^. Leisure-time physical activity was measured using four self-report questions from which metabolic equivalent tasks (MET) were calculated. MET hours per week were included as a continuous variable. Smoking was classified as current smoking and non-smoking. Alcohol problems were measured using the CAGE–scale, which asks four questions, namely cutting down on alcohol, annoyed by criticism, feeling guilty, and in need of an eye-opener [37]. The CAGE–scale was used as a continuous variable.

### Statistical methods

The associations between parental education and childhood adversities and disability retirement were analysed using Cox regression analysis. Hazard ratios (HR) and their 95% confidence intervals (CI) were calculated. Age of the participant was used as the time scale in the Cox proportional hazards models. Proportionality assumptions were tested and met, except for the model concerning parental alcohol problems and disability retirement due to musculoskeletal diseases. In order to meet the proportionality assumption, a stratified analysis for those under 50 years of age and those aged 50 or over was performed and reported on.

Women and men were pooled in the analyses. We did not have enough statistical power to perform separate analyses due to the relatively small amount of men. Interactions for sex were tested and only one significant interaction was found, namely the association between childhood illness and disability retirement due to mental diagnosis (p<0.1). Base models adjusted for sex and age were fitted for disability retirement by each childhood variable. Then other covariates were added one by one to the models: first education; next marital status; then working conditions, and finally weight and health behaviours. The SAS statistical program version 9.4 and R statistical software were used to perform the analyses.

## Results

Educational attainment was higher among the participants’ fathers than mothers, but low education was the most common level for both parents. (Table 1) Prevalence of childhood adversities varied between 5% and 20%, parental alcohol problems and economic difficulties in the family being the most common exposures. The number of disability retirement events varied by parental education and childhood adversities. Disability retirement due to musculoskeletal diseases was more common than disability retirement due to mental disorders.

**Table 1:** Descriptive information on childhood conditions and disability retirement.

	Distribution of childhood conditions		Incidence of disability retirement (n of cases during follow-up)		
	n	%	All causes	Musculoskeletal diseases	Mental disorders
Maternal education					
High	305	5	24	4	14
Mid-level	1273	21	123	40	45
Low	4357	73	614	274	150
Paternal education					
High	619	11	47	10	23
Mid-level	1508	26	168	59	58
Low	3762	64	535	240	127
Childhood illness					
No	5093	93	619	264	169
Yes	397	7	71	24	21
Parental divorce					
No	4857	89	596	252	158
Yes	627	11	81	32	25
Parental death					
No	4781	87	589	249	160
Yes	705	13	85	31	27
Parental mental illness					
No	5134	95	622	266	161
Yes	284	5	57	14	32
Parental alcohol problems					
No	4444	80	537	223	140
Yes	1110	20	169	75	54
Economic difficulties in family					
No	4545	82	527	221	137
Yes	995	18	174	78	51
Bullying					
No	5012	92	594	254	157
Yes	456	8	88	34	30

Both low maternal (HR 1.74, 95% CI 1.16–2.62) and paternal (1.86, 1.38–2.51) education were associated with all-cause disability retirement after adjustment for age and sex. (Table 2) Paternal mid-level educational attainment was associated with disability retirement in comparison to paternal high education. Adjustment for own education abolished these associations. Economic difficulties in the childhood family were associated with an increased risk of all-cause disability retirement (1.48, 1.25–1.76). The association was attenuated after adjustment for own education, marital status, working conditions, and weight and health behaviours. Childhood illness increased the risk of all-cause disability retirement even after all adjustments (1.44, 1.12–1.84), although the risk slightly attenuated after adjustment for working conditions, and weight and health behaviours. Parental divorce and parental death were not associated with all-cause disability retirement, whereas parental mental illness increased the risk (1.68, 1.28–2.20), which remained elevated after adjustment for own education, marital status, working conditions, and weight and health behaviours. Parental alcohol problems increased the risk of all-cause disability retirement (1.32, 1.11–1.57) and the association attenuated after adjustment for education, marital status and working conditions but disappeared in the fully-adjusted model. Bullying at school or by peers increased the risk of all-cause disability retirement and this association remained, although it was somewhat attenuated after the adjustments (1.55, 1.24–1.95).

**Table 2:** Associations between childhood conditions and all-cause disability retirement. Hazard ratios and their 95% confidence intervals.

	Sex	+ own education	+ marital status	+ working conditions	+ weight + health behaviours*
Maternal education					
High	1.00	1.00	1.00	1.00	1.00
Mid-level	1.25 (0.81–1.94)	1.05 (0.67–1.62)	1.05 (0.67–1.63)	1.03 (0.66–1.60)	1.02 (0.66–1.59)
Low	1.74 (1.16–2.62)	1.25 (0.82–1.89)	1.25 (0.83–1.89)	1.22 (0.81–1.85)	1.21 (0.80–1.83)
Paternal education					
High	1.00	1.00	1.00	1.00	1.00
Mid-level	1.50 (1.09–2.08)	1.21 (0.88–1.68)	1.21 (0.87–1.68)	1.19 (0.85–1.65)	1.19 (0.86–1.65)
Low	1.86 (1.38–2.51)	1.31 (0.96–1.79)	1.30 (0.96–1.77)	1.26 (0.93–1.72)	1.28 (0.94–1.74)
Economic difficulties in the family	1.48 (1.25–1.76)	1.37 (1.16–1.63)	1.36 (1.14–1.62)	1.31 (1.10–1.56)	1.24 (1.04–1.47)
Childhood illness	1.53 (1.20–1.95)	1.56 (1.22–1.99)	1.54 (1.20–1.97)	1.48 (1.16–1.90)	1.44 (1.12–1.84)
Parental divorce	1.11 (0.88–1.41)	1.00 (0.79–1.26)	0.99 (0.78–1.25)	1.00 (0.79–1.26)	0.96 (0.76–1.22)
Parental death	1.02 (0.81–1.28)	0.93 (0.74–1.17)	0.93 (0.74–1.17)	0.92 (0.73–1.16)	0.89 (0.71–1.12)
Parental mental illness	1.68 (1.28–2.20)	1.71 (1.30–2.25)	1.69 (1.28–2.21)	1.62 (1.23–2.12)	1.61 (1.23–2.12)
Parental alcohol problems	1.32 (1.11–1.57)	1.25 (1.05–1.49)	1.25 (1.05–1.48)	1.23 (1.04–1.47)	1.19 (1.00–1.42)
Bullying	1.76 (1.41–2.20)	1.71 (1.37–2.14)	1.69 (1.35–2.11)	1.63 (1.30–2.05)	1.55 (1.24–1.95)

*health behaviours included physical activity, problem drinking and smoking

Low maternal education showed a high risk of disability retirement (4.44, 1.66–11.92) due to musculoskeletal diseases, which nevertheless disappeared after adjustment for own education. (Table 3) Low (3.81, 2.02–7.17) and mid-level (2.48, 1.27–4.84) paternal education increased the risk of disability retirement due to musculoskeletal diseases compared to high paternal education. Adjustment for own education abolished the association with mid-level education and attenuated the association with low education. Economic difficulties in the family were associated with an increased risk of disability retirement due to musculoskeletal diseases (1.55, 1.20–2.00). This association was attenuated after adjustment for education and abolished after further adjustment for working conditions. Childhood illness, parental death or divorce, and parental mental illness were not associated with disability retirement due to musculoskeletal diseases. Parental alcohol problems increased the risk among those aged 50 or over but the association was abolished after adjustments. Having been bullied increased risk of disability retirement due to musculoskeletal diseases and this association remained after all adjustments (1.46, 1.01–2.09).

**Table 3:** Associations between childhood conditions and disability retirement due to musculoskeletal diseases.

	Sex	+ own education	+ marital status	+ working conditions	+ weight + health behaviours*
Maternal education					
High	1.00	1.00	1.00	1.00	1.00
Mid-level	2.39 (0.85–6.67)	1.64 (0.59–4.60)	1.64 (0.58–4.58)	1.52 (0.54–4.28)	1.44 (0.51–4.04)
Low	4.44 (1.66–11.92)	2.33 (0.86–6.28)	2.32 (0.86–6.26)	2.10 (0.78–5.69)	2.00 (0.74–5.40)
Paternal education					
High	1.00	1.00	1.00	1.00	1.00
Mid-level	2.48 (1.27–4.84)	1.61 (0.82–3.17)	1.62 (0.83–3.18)	1.56 (0.79–3.06)	1.48 (0.75–2.91)
Low	3.81 (2.02–7.17)	1.95 (1.03–3.72)	1.97 (1.04–3.75)	1.83 (0.96–3.48)	1.73 (0.90–3.29)
Economic difficulties in family	1.55 (1.20–2.00)	1.38 (1.07–1.79)	1.39 (1.07–1.80)	1.31 (1.01–1.71)	1.25 (0.96–1.63)
Childhood illness	1.23 (0.81–1.87)	1.28 (0.84–1.94)	1.28 (0.85–1.95)	1.23 (0.81–1.88)	1.22 (0.80–1.86)
Parental divorce	1.10 (0.76–1.59)	0.92 (0.64–1.33)	0.92 (0.64–1.33)	0.93 (0.64–1.35)	0.91 (0.63–1.32)
Parental death	0.86 (0.59–1.24)	0.74 (0.51–1.08)	0.74 (0.51–1.08)	0.72 (0.49–1.04)	0.68 (0.47–1.00)
Parental mental illness	0.96 (0.56–1.65)	1.01 (0.59–1.73)	1.02 (0.59–1.74)	0.96 (0.56–1.65)	1.00 (0.58–1.71)
Parental alcohol problems					
Employees under 50 years at baseline	1.40 (0.87–2.25)	1.31 (0.81–2.10)	1.29 (0.80–2.08)	1.27 (0.79–2.05)	1.36 (0.84–2.22)
Employees 50 years or over at baseline	1.48 (1.08–2.02)	1.34 (0.98–1.84)	1.35 (0.99–1.85)	1.36 (0.99–1.86)	1.32 (0.97–1.82)
Bullying	1.68 (1.17–2.40)	1.62 (1.13–2.32)	1.63 (1.14–2.34)	1.52 (1.06–2.18)	1.46 (1.01–2.09)

*health behaviours included physical activity, problem drinking and smoking

Parental education, parental death and divorce were not associated with disability retirement due to mental disorders. (Table 4) Economic difficulties in the family were associated with an increased risk (1.71, 1.24–2.36). Adjustment for own education had no effect on the association, whereas further adjustment for marital status, working conditions, and weight and health behaviours attenuated it. Parental mental illness was strongly associated with disability retirement due to mental disorders even after all adjustments (3.04, 2.07–4.47). Parental alcohol problems were associated with an increased risk (1.55, 1.15–2.15). Education and marital status had no effect on the association, which was somewhat attenuated after adjustment for working conditions and abolished after further adjustment for weight and health behaviours. Having been bullied increased the risk of disability retirement due to mental disorders (2.16, 1.46–3.20). The association was slightly attenuated after adjustment for covariates but remained in the fully adjusted models.

**Table 4:** Associations between childhood conditions and disability retirement due to mental disorders. Hazard ratios and their 95% confidence intervals.

	Sex	+own education	+ marital status	+ working conditions	+ weight + health behaviours*
Maternal education					
High	1.00	1.00	1.00	1.00	1.00
Mid-level	0.79 (0.43–1.44)	0.77 (0.42–1.40)	0.77 (0.42–1.40)	0.77 (0.42–1.40)	0.78 (0.43–1.43)
Low	0.76 (0.44–1.32)	0.73 (0.42–1.29)	0.73 (0.41–1.27)	0.73 (0.42–1.28)	0.74 (0.42–1.30)
Paternal education					
High	1.00	1.00	1.00	1.00	1.00
Mid-level	1.05 (0.65–1.70)	1.01 (0.62–1.64)	0.98 (0.60–1.61)	0.97 (0.59–1.58)	1.00 (0.61–1.65)
Low	0.92 (0.59–1.44)	0.89 (0.56–1.42)	0.87 (0.54–1.38)	0.86 (0.54–1.37)	0.90 (0.56–1.44)
Economic difficulties in family	1.71 (1.24–2.36)	1.71 (1.24–2.37)	1.64 (1.18–2.26)	1.57 (1.14–2.18)	1.46 (1.05–2.03)
Childhood illness	1.65 (1.05–2.59)	1.65 (1.05–2.60)	1.56 (0.99–2.45)	1.50 (0.96–2.37)	1.41 (0.89–2.22)
Parental divorce	1.23 (0.81–1.88)	1.22 (0.80–1.87)	1.16 (0.76–1.78)	1.18 (0.77–1.81)	1.13 (0.73–1.73)
Parental death	1.23 (0.82–1.85)	1.23 (0.82–1.85)	1.23 (0.81–1.85)	1.22 (0.81–1.84)	1.19 (0.79–1.79)
Parental mental illness	3.60 (2.46–5.26)	3.61 (2.47–5.27)	3.41 (2.33–4.99)	3.23 (2.20–4.73)	3.04 (2.07–4.47)
Parental alcohol problems	1.57 (1.15–2.15)	1.55 (1.13–2.12)	1.53 (1.12–2.10)	1.51 (1.10–2.06)	1.36 (0.99–1.87)
Bullying	2.16 (1.46–3.20)	2.12 (1.43–3.14)	1.96 (1.32–2.90)	1.93 (1.30–2.86)	1.82 (1.22–2.70)

*health behaviuors included physical activity, problem drinking and smoking

## Discussion

This study aimed to examine the associations of parental education and childhood adversities with midlife disability retirement. Parental education was associated with all-cause disability retirement and disability retirement due to musculoskeletal diseases but not with disability retirement due to mental disorders. The associations however mainly disappeared after adjustment for own education. Of the childhood adversities economic difficulties in the family, parental alcohol problems and having been bullied at school or by peers were associated with all the studied diagnosis groups of disability retirement, whereas parental death and divorce made no contributions. Parental mental illness and childhood illness were associated with an increased risk of all-cause disability retirement and disability retirement due to mental disorders, the association between parental mental illness and disability retirement due to mental disorders being particularly strong. The contributions of childhood adversities to disability retirement mainly remained after adjustment for own education but were attenuated after further adjustment for working conditions, and weight and health behaviours.

Some previous studies have also examined the contributions of childhood socioeconomic position to disability retirement and have produced somewhat inconclusive results. A UK study focusing on middle-aged adults measured childhood socioeconomic position by parental occupational class and found no association [32] whereas a Swedish study examining disability retirement at a relatively young age, namely until the age of 43, found that father’s low occupational class was associated with an increased risk [24]. A Norwegian study examined disability retirement at a young age and found that parental education only had an effect on low educated men [26]. Our study suggests that the contribution of childhood socioeconomic position may vary according to the diagnosis behind disability retirement, as childhood socioeconomic position was associated with all-cause disability retirement and disability retirement due to musculoskeletal diseases but not with disability retirement due to mental disorders. Studies of the different diagnoses behind disability retirement are scarce, but a Finnish study of a birth cohort of young adults found no strong associations between mother’s and father’s education and disability retirement due to mental disorders before the age of 25 [28]. Swedish studies have found, partly in contrast to our study, that father’s occupation was associated with all-cause disability retirement, alcohol-related disability retirement diagnosis and other psychiatric diagnoses but not with psychosis diagnosis [24–25]. Compared to our study, the participants were younger, 43 years old at the most, and thus the mental disorders behind disability retirement might be different to those in our study on older employees. Most mental diseases begin during the first three decades of life [38].

The pathways behind the association between childhood socioeconomic position and disability retirement have not been widely examined. In our study own education largely abolished the association between parental education and disability retirement. This effect might be partly due to limited statistical power in the analysis, but the finding suggests that the contribution of parental education to disability retirement is mostly mediated through one’s own education. Previous research has shown that childhood socioeconomic position is associated with adult socioeconomic position [39], which is a key determinant of health and has been associated with disability retirement [19]. Our study adds to prior evidence by examining both paternal and maternal education and their contributions to disability retirement, which were rather similar.

In addition to childhood socioeconomic position different childhood adversities were examined. Parental death and divorce showed no associations with disability retirement. The results of previous studies have provided inconclusive results. In line with our findings, a British study found no association with parental divorce [32], whereas in a US study parental divorce was associated with self-reported inability to work [40]. In a Finnish study focusing on young adults, parental divorce was associated with disability retirement due to mental disorders before the age of 25 [28]. In Swedish studies, not being brought up by both parents was associated with an increased risk of all-cause disability retirement and disability retirement due to alcohol-related diagnoses at a relatively young age [24]. Furthermore, parental divorce increased the risk of disability retirement due to psychosis, alcohol-related diagnosis and other psychiatric diagnosis [25]. Of the studied childhood adversities, economic difficulties in the childhood family, parental alcohol problems and having been bullied at school or by peers made the most widespread contributions to disability retirement, as they were associated with all the disability retirement diagnoses studied. To our knowledge, economic difficulties in the childhood family and having been bullied have not been examined previously. However, a US study found that living with someone with substance abuse problems was associated with self-reported work inability [40], whereas a Swedish study found no association between fathers’ alcohol consumption and all-cause disability retirement and alcohol-related disability retirement among young men [41]. In line with a Norwegian study [26], childhood illness was associated with an increased risk of all-cause disability retirement, suggesting that the origins of a disabling disease may lay in childhood or may have predisposed the individual to adulthood disease. Parental mental illness increased the risk of all-cause disability retirement and was strongly associated with disability retirement due to mental disorders. Our study thus supports previous findings regarding how parental mental health contribute to offspring mental health [42] and disability retirement due to mental disorders [28].

To summarize, the contributions of various childhood adversities have somewhat varied in different studies. In addition, some studies have examined the contribution of the accumulation of adversities instead of single adversities [15]. The pathways from childhood adversities to disability retirement might vary according to adversity. In our study, childhood illness was associated with an increased risk of disability retirement which might be partly due to childhood illness contributing to adult health. However, as the studied employees were already in midlife, it is likely that most of the childhood contribution to disability retirement was indirect, for example childhood adversities affecting determinants of adult health and disability retirement. With the exception of the association between parental alcohol problems and disability retirement due to musculoskeletal diseases, the contribution of childhood adversities was not abolished after adjustment for own education, which suggests that in our study adult socioeconomic position was not an important mediator between childhood adversities and disability retirement. Marital status only had a small effect on the associations and this effect was limited to disability retirement due to mental disorders. These results suggest that childhood adversities might contribute to adult marital status which is a known determinant of adult mental health [43]. Working conditions and adult weight and health behaviours all somewhat contributed to the associations, implying that childhood adversities might influence adult jobs and health behaviours, which in turn may act as determinants of disability retirement. Bullying at school or by peers made a widespread contribution to all studied disability retirement diagnoses and the associations remained after all adjustments. It might thus be that bullying that occurs during a vulnerable time period has a long-lasting effect on adult health and work ability. The association between parental mental illness and disability retirement due to mental disorders was strong even after all adjustments, also suggesting long-term effects of childhood exposures. As regards mental illness, however, hereditary features also play a role and should be taken into account. Overall, the pathways from childhood to disability retirement are complex and vary according to different childhood exposures and disability retirement diagnoses. There is a likely network of associations, some of which might be due to the accumulation of exposures and others due to exposures at vulnerable periods.

The advantages of the study include a relatively large data set, prospectively followed disability retirement after reporting parental education and childhood adversities, and the fact that we were able to control for several sociodemographic, health and work-related covariates. The data on disability retirement and their diagnosis were register-based and thus reliable. The follow-up period was long, but by definition childhood circumstances did not change during this time. The data on parental education and childhood adversities were self-reported and elicited retrospectively, and thus might be biased by the current situation. It might be that questions with clear-cut answers such as parental death are less susceptible to bias whereas questions such as being bullied might be more affected by mood and other conditions during the time of the survey. A study on the accuracy of adult recall of childhood socioeconomic position has suggested that studies based on adult recall are likely to underestimate real effects [44], whereas a review on adult recall of childhood adversities has reported that several studies have shown some bias [45]. As the studied employees were already in midlife and employed, it might be that the associations are underestimated, as those with more childhood adversities and poorer health might have retired or otherwise selected out of the workforce before midlife. On the other hand, we were specifically interested in midlife employees rather than the young. Our measure of childhood adversities did not include abuse or neglect of children, which has been associated with adult mental and somatic morbidity in previous research [46]. Thus, it is likely that the study lacked some of childhood’s contribution to adult disability retirement.

In conclusion, the study underlines the importance of childhood for adult work ability. The findings of the study that parental education and childhood adversities contribute to disability retirement even in midlife, expand on those of previous research. Childhood was associated with all-cause disability retirement and with disability retirement due to musculoskeletal and mental diagnoses, but the pathways and contributions of parental education and diverse childhood adversities were different. Low parental education increased the risk of all-cause disability retirement and disability retirement due to musculoskeletal diseases, and the effect seemed to be mediated by own education. Economic difficulties in the childhood family, parental alcohol problems and having been bullied at school or by peers showed the most widespread contributions, whereas the association between parental mental illness and disability retirement due to mental disorders was particularly strong. Policy actions investing in children and improving their living conditions might promote health and work ability in midlife and lead to economic savings in the long run. The children of parents suffering from mental disorders should be considered risk group for disability retirement.
